# Intrathecal baclofen withdrawal syndrome- a life-threatening complication of baclofen pump: A case report

**DOI:** 10.1186/1472-6904-4-6

**Published:** 2004-08-09

**Authors:** Imran Mohammed, Asif Hussain

**Affiliations:** 1 Department of Internal Medicine, Mercy Hospital of Pittsburgh, 1400 Locust Street, Pittsburgh, Pennsylvania 15219, United States of America; 2 Department of Pharmaceutical Chemistry, Jamia Hamdard University, Hamdard Nagar, New Delhi 110062, India

## Abstract

**Background:**

Intrathecal baclofen pump has been used effectively with increasing frequency in patients with severe spasticity, particularly for those patients who are unresponsive to conservative pharmacotherapy or develop intolerable side effects at therapeutic doses of oral baclofen. Drowsiness, nausea, headache, muscle weakness, light-headedness and return of pretreatment spasticity can be caused by intrathecal pump delivering an incorrect dose of baclofen. Intrathecal baclofen withdrawal syndrome is a very rare, potentially life-threatening complication of baclofen pump caused by an abrupt cessation of intrathecal baclofen.

**Case presentation:**

A 24-year-old man with a past medical history of cerebral palsy and spastic quadriparesis developed hyperthermia, disseminated intravascular coagulation, rhabdomyolysis, acute renal failure and multisystem organ failure leading to a full-blown intrathecal baclofen withdrawal syndrome. Intrathecal baclofen pump analysis revealed that it was stopped due to some programming error. He was treated effectively with supportive care, high-dose benzodiazepines and reinstitution of baclofen pump.

**Conclusion:**

The episodes of intrathecal baclofen withdrawal syndrome are mostly caused by preventable human errors or pump malfunction. Educating patients and their caregivers about the syndrome, and regular check-up of baclofen pump may decrease the incidence of intrathecal baclofen withdrawal syndrome. Oral baclofen replacement may not be an effective method to treat or prevent intrathecal baclofen withdrawal syndrome. Management includes an early recognition of syndrome, proper intensive care management, high-dose benzodiazepines and prompt analysis of intrathecal pump with reinstitution of baclofen.

## Background

Baclofen is a gamma-aminobutyric acid (GABA) analog that has inhibitory effects on spinal cord reflexes and brain. Intrathecal baclofen (ITB) therapy consists of long-term delivery of baclofen to the intrathecal space. Intrathecal baclofen has been used to treat spasticity due to cerebral palsy, brain or spinal cord injury, multiple sclerosis, dystonia, stroke and stiff-man syndrome, particularly for those patients who are unresponsive to conservative pharmacotherapy or develop intolerable side effects at therapeutic doses of oral baclofen [1]. Side effects such as drowsiness, nausea, headache, muscle weakness and light-headedness can occur as a result of the pump delivering an incorrect dose of baclofen. Sudden cessation of ITB administration can cause mild symptoms like reappearance of baseline level of spasticity associated with pruritis, anxiety and disorientation [2]. These mild symptoms represent "loss of drug effect". All patients experience "loss of drug effect" when ITB is discontinued, only a small (but unknown) proportion of patients develop a full-blown potentially life-threatening withdrawal syndrome. We report a case of ITB withdrawal syndrome developing hyperthermia, severe spasticity, disseminated intravascular coagulation, rhabdomyolysis, acute renal failure and multisystem organ failure.

## Case presentation

A 24-year-old man was admitted to our intensive care unit (ICU) with a possible diagnosis of seizure disorder and sepsis. He had a past medical history of cerebral palsy and spastic quadriparesis. Three years ago, he had an ITB pump implanted for spasticity refractory to the high doses of oral baclofen. He had a significant improvement in spasticity, social and functional capacity in the past three years.

Later, he developed some disorientation and increased spasticity. He was taken to a local physician who prescribed oral baclofen (120 mg daily in four divided doses) for his increased spasticity. He also advised him to have his ITB pump checked immediately. The following day, his spasticity increased even after taking oral baclofen. He developed multiple seizures and respiratory distress in the next 24-hour period. Subsequently, he was admitted in a local hospital where he was orally intubated and transferred to our ICU for aggressive management.

On presentation, his temperature was 104.6°F (40.3°C), heart rate 127 beats per minute, and the blood pressure was 85/45 mm/Hg. His ventilator settings were: assist-control ventilation mode; respiratory rate, 15 breaths per minute; tidal volume, 650 mL; positive end expiratory pressure (PEEP), 5 cm H_2_O; and FiO_2_, 60%. His spontaneous respiratory rate was 18 breaths per minute and an oxygen saturation of 100% was noted on pulse oximetry. In the local hospital, he was documented to have a high fever of 107°F (41.6°C) and he had received intravenous lorazepam, phenytoin, pantoprazole, piperacillin/tazobactem and dopamine. On physical examination, neurologically he was unconscious with decerebrate posturing and his Glasgow coma scale was 6. He had an absent corneal and gag reflexes. He was moving all four limbs in response to noxious stimuli. He was also noted to have an extreme spasticity in all four limbs. Lung examination revealed decreased breath sounds in the left lower base. Cardiac examination was unremarkable. He had a palpable baclofen pump on abdominal wall and bowel sounds were heard. The differential diagnoses were septic shock, meningitis, neuroleptic malignant syndrome and malignant hyperthermia.

The initial laboratory results showed serum creatinine phosphokinase (CPK) 5250 U/L (Normal, 25–235 U/L) and CPK-MB fraction 12.1 ng/ml (Normal, 0.5–6.3 U/L). Serum chemistry revealed sodium 142 mmol/L, potassium 5.1 mmol/L, chloride 120 mmol/L, bicarbonate 13 mmol/L, and creatinine 2.1 mg/dl. Hemogram showed white blood cell count 12.2 K/UL, hemoglobin 16.5 g/dl and platelet count 9 K/UL (Normal, 130–400 K/UL). Liver function test showed aspartate aminotransferase (ALT) 1128 U/L, alanine aminotransferase (AST) 1140 U/L, alkaline phophatase 90 U/L, total bilirubin 1.2 mg/dl, conjugated bilirubin 0.7 mg/dl, prothrombin time 20.2 seconds (Normal, 10–12.5 seconds), and INR 2.0 (Normal, 0.9–1.1). Blood and urine cultures were obtained. Chest radiograph was normal. A computed tomography (CT) scan of the chest revealed atelectasis of the left lung base. His CT scan of head did not show any acute infarct or bleeding. His initial management included intravenous fluids, norepinephrine, platelet transfusion, phenytoin, propofol and broad-spectrum antibiotics (vancomycin, ceftriaxone) for suspected meningitis and septic shock. He received intravenous lorazepam (4–8 mg every four hours) for his spasticity. Next day, his spasticity improved and an ITB specialist investigated his baclofen pump. His baclofen pump analysis revealed that it was stopped due to some programming error, which was restarted at a previously prescribed baclofen rate (260 μg/day).

On third hospital day, his serum CPK was 15,878 U/L, AST was 2566 U/L, ALT was 2993 U/L, while CPK-MB fraction came down to 3.4 ng/ml. His urine output decreased (<400 ml/ day) and serum creatinine increased in the range of 5–6 mg/dl. Later, he was hemodialyzed few times during the course of hospitalization due to acute renal failure. His echocardiogram showed left ventricular ejection fraction of 20–25% and severe global hypokinesis. His electroencephalogram did not reveal any epileptogenic activity. He developed full-blown multisystem organ failure with an evidence of shock liver, renal failure, respiratory failure, disseminated intravascular coagulation and myocardial depression. His nutrition was started on nasogastric tube feedings, and proper ventilator care was taken through a tracheostomy tube. His serum baclofen obtained at the time of admission was less than 0.02 μg/ml (Expected values, 0.08–0.4 μg/ml). After a three-week course of aggressive management in ICU, he was weaned off from the ventilator and his multiple organ shock resolved. At a six-month follow-up, he was observed in a nursing home with his baseline functional, social, and family activities.

## Discussion

Intrathecal baclofen provides an effective improvement in the spasticity of patients whose spasticity is not sufficiently managed by oral baclofen or other oral anti-spastic medications [1]. Regardless of the cause of spasticity (cerebral or spinal), anti-spastic effects of baclofen occur at spinal level. Poor response of oral baclofen in many patients can be explained by the fact that the spinal cord represents only about 2% mass of the brain, and receives proportionately a lower blood flow as a fraction of cardiac output. Therefore, cerebral side effects often occur before therapeutic anti-spastic effects of oral baclofen are observed. A programmable ITB pump provides a direct, pattern-controlled delivery of baclofen to the spinal cord. Precise delivery of the ITB pump yields better spasticity reduction at 1000 times lower than the doses of oral baclofen. In addition, the adverse effects are minimized as compared with oral baclofen. ITB provides reduced tone, spasms and pain, improves speech, mobility, sleep quality and bladder control, with a response rate up to 97% in adults and children [3,4]. The ITB pump is approximately 3 inches wide and 1 inch thick. It is surgically implanted in the subcutaneous tissue of anterior abdominal wall. Baclofen is delivered via a silicone rubber catheter into the lumbar subarachanoid space. The ITB pump delivers approximately 100–900 μg/day of baclofen, and it is titrated for the desired clinical response. It is also equipped with an alarm that signals low volume, low battery, or malfunction. Nine years ago, catheter or pump-related malfunction that leads to an overdose or withdrawal has been reported in 40 % of the patients with ITB pump [5]. Catheter system, operative techniques and programming system of the ITB pump have now been improved significantly to reduce the incidence of an overdose or withdrawal.

The precise mechanism of action of baclofen as a muscle relaxant and anti-spasticity agent is not fully understood. Baclofen inhibits both monosynaptic and polysynaptic reflexes at the spinal cord level [6], possibly by decreasing excitatory neurotransmitter release from primary afferent terminals, although actions at supraspinal sites may also contribute to its clinical effects. Baclofen also causes enhancement of vagal tone and inhibition of mesolimbic and nigrostriatal dopamine neurons (directly or via inhibiting substance P) [7]. Baclofen is a structural analog of the inhibitory neurotransmitter GABA, and may exert its effects by stimulation of the GABA_B _receptor to cause muscle relaxation. Baclofen reduces increased muscle tone, Babinski sign, tendon reflexes, ankle clonus and sometimes decreases muscle force.

Long-term ITB infusion causes down-regulation of GABA_B _receptors in the CNS and spinal cord. Down-regulation of GABA_B _receptors accounts for the decreased sensitivity to the baclofen over time. Although GABA_B _receptors are down-regulated, it is the baclofen itself that causes increased inhibitory tone in the CNS and spinal cord [8]. Therefore, abrupt ITB withdrawal results in a predominance of excitatory effects and simulates other conditions that are associated with CNS hyperexcitability and severe spasticity. Sudden cessation of ITB administration can cause mild symptoms like reappearance of baseline level of spasticity associated with pruritis, anxiety and disorientation. These mild symptoms represent "loss of drug effect". All patients experience "loss of drug effect" when ITB is discontinued. However, more severe symptoms like hyperthermia (109.4°F), myoclonus, seizures [9-12], rhabdomyolysis, disseminated intravascular coagulation, multisystem organ failure [10], cardiac arrest, coma and death [9,12,13] have been well reported, and represents a full-blown life-threatening ITB withdrawal syndrome. Food and drug administration (FDA) of USA has included a drug label warning for baclofen withdrawal syndrome in April 2002 [1,13]. Differential diagnoses include malignant hyperthermia, neuroleptic-malignant syndrome, autonomic dysreflexia, sepsis and meningitis. ITB withdrawal syndrome has been fatal in some cases. Six patients have died out of 27 cases reported to FDA [13]. Most reported episodes of ITB withdrawal were caused by preventable human errors or oversights. However, catheter dislodgement, catheter migration and kinks, and other catheter-related issues might be more common than pump-related malfunctions [1,14]. Close attention to pump refilling and programming procedures may reduce the incidence of ITB withdrawal syndrome.

Benzodiazepines are helpful in controlling spasticity and seizures during ITB withdrawal syndrome [1,10]. Benzodiazepines activate central receptors and GABA_A _receptors of spinal cord by different mechanisms [1]. Therefore, ITB induced down-regulation of GABA_B _receptors do not interfere with benzodiazepine's mechanism of action. During a planned removal of ITB pump due to infection or other causes, premedication with high doses of benzodiazepines and augmented oral baclofen is usually administered in the hospitals to prevent spasticity. Similarly, high doses of oral baclofen is also tried in some cases of ITB withdrawal syndrome [15,16]. But failure of high doses of oral baclofen (80 mg three times daily) have been reported recently [17]. High doses of oral baclofen may not be adequate to treat or prevent ITB withdrawal because of down-regulation of central GABA_B _receptors due to chronic ITB administration. Moreover, it has been suggested that it may take many hundreds of grams of oral baclofen to achieve a therapeutic baclofen level in the cerebrospinal fluid, compared to the patients who had effective spasticity control with an ITB pump [17]. Although, our patient received oral baclofen (120 mg daily in four divided doses) initially but these doses may be low enough to prevent ITB withdrawal syndrome. Failure of high doses of oral baclofen suggests that resumption of GABA_B _receptor agonist by prompt restoration of ITB pump and proper supportive care might be the best treatment. Similarly, high-dose benzodiazepines may be effective because of similar mechanism of action on widespread CNS GABA_A _receptors. High-dose benzodiazepines could be an initial life saving strategy even before analysis and restoration of ITB pump is achieved, or in cases, where resumption of ITB administration is not as simple as correcting a programming error.

Intrathecal baclofen bolus is appropriate, but due to the risk of inadvertent overdose, an experienced physician should immediately perform reinstitution of ITB pump. We had restarted ITB in our patient at a previously prescribed dose. However, a much higher dose of baclofen could have safely been given to overcome the spasticity since the patient was in an ICU. High-dose dantrolene (a direct muscle relaxant that acts on sarcoplasmic reticulum of skeletal muscle) has been tried to reduce spasticity and fever in ITB withdrawal syndrome [18]. Reduction in fever may be due to cessation of repetitive and thermogenic contractions of muscle fibers. It is unlikely that dantrolene has any GABA agonistic effects, and administration of dantrolene may not be accompanied by any correction in anomalies of CNS functions. Therefore, seizures, autonomic instability and death may occur in ITB withdrawal syndrome even after controlling spasticity with dantrolene. Cyproheptadine (a non-selective serotonin antagonist) has also been used postulating that ITB withdrawal may be a form of serotonergic syndrome that occurs from the loss of GABA_B _receptor-mediated presynaptic inhibition of serotonin [19]. We did not consider dantrolene or cyproheptadine in our patient due to lack of sufficient clinical support in the treatment of ITB withdrawal syndrome. There was a three-day period of delay in diagnosing the ITB withdrawal syndrome leading to deterioration and multisystem organ failure. However, our patient had a successful recovery in response to restoration of baclofen pump and adequate intensive care management.

## Conclusions

Baclofen withdrawal syndrome is a potentially life-threatening complication of intrathecal baclofen pump. Empty pump reservoir, catheter leaks or displacement, pump malfunction, programming error and refill of pump with improper drug concentration are the possible mechanisms which could lead to an ITB withdrawal syndrome. Regular check-up of the ITB pump by a specialist, educating patients and their caregivers may decrease the incidence of ITB withdrawal syndrome. Oral baclofen replacement may not be an effective method to treat or prevent ITB withdrawal syndrome. Early recognition of syndrome, high-dose benzodiazepines, prompt analysis of the ITB pump with reinstitution of baclofen, and proper intensive care management are mainstays for the management of ITB withdrawal syndrome.

## List of abbreviations

GABA -gamma amino butyric acid, ITB – intrathecal baclofen, CPK – creatinine phosphokinase, ALT – aspartate aminotransferase, AST – alanine aminotransferase, CT – computed tomography.

## Competing interests

None declared.

## Authors' contributions

IM: Direct patient care, article conception, critical and extensive revision of article for important intellectual content, review and drafting of the original article. AH: Literature search, case review and summary, drafting of the original article. Both authors read and approved the final manuscript and contributed equally to the manuscript.

## Pre-publication history

The pre-publication history for this paper can be accessed here:


